# Did Down‐Regulated Instincts Enable Human Gene‐Culture Coevolution?

**DOI:** 10.1002/evan.70015

**Published:** 2025-08-14

**Authors:** Gerald E. Loeb

**Affiliations:** ^1^ Alfred E. Mann Dept. of Biomedical Engineering University of Southern California

**Keywords:** behavior, cultural evolution, genetic evolution, hormones, Instincts, pheromones

## Abstract

The unique intellectual and cultural attributes of *Homo sapiens* that arose during the Middle Stone Age are often ascribed to positive evolutionary development of novel physical or personality traits, but attempts to correlate cultural with genetic evolution have been unsuccessful. Humans are also unique, however, in their ability to ignore or override hormonal and pheromonal instincts that define the social structures and behaviors of other animals. Humans can rapidly invade new environments because they invent rather than inherit such behaviors, which cumulatively we call a culture. Downregulation of instincts makes the invention and learning of cultures necessary, which imposes both an opportunity and a burden on individuals and societies. Cultural evolution enables human societies to invent, promulgate, compete and evolve their social structures in a generation or two rather than the hundreds of generations required for significant genetic evolution. Nevertheless, residual instincts may conflict with and delimit novel cultures and their social structures.

## 1 Introduction

Modern humans have intellectual and cultural attributes that are sufficiently distinct from all other animal species that we have little difficulty thinking of ourselves as *sui generis*. Science only recently rejected the notion that humans were a divine creation in favor of the reality that *Homo sapiens* represents just another, relatively small, step in the genetic evolution of primates [1]. This leads to the question of how the qualitatively distinctive attributes arose – the problem of human uniqueness [2]. Many physical enhancements to the body and brain of humans have been identified but they are more quantitative and gradual, with precedents in other hominins, primates and mammals. Furthermore, the physical and genetic speciation of *Homo sapiens* appears to have been largely complete at the beginning of the Middle Stone Age (MSA) about 300,000 years ago [3], before humans were particularly unique. Attempts to identify an orderly progression of human cultural evolution that could be associated with the genetic appearance of socially stabilizing traits during the MSA have been unsuccessful [4]. Furthermore, traits such as empathy, altruism and fairness have been well‐documented in nonhuman primates [5, 6] but they have not given rise to the large‐scale socioeconomic organizations of humans.

**Figure 1:**
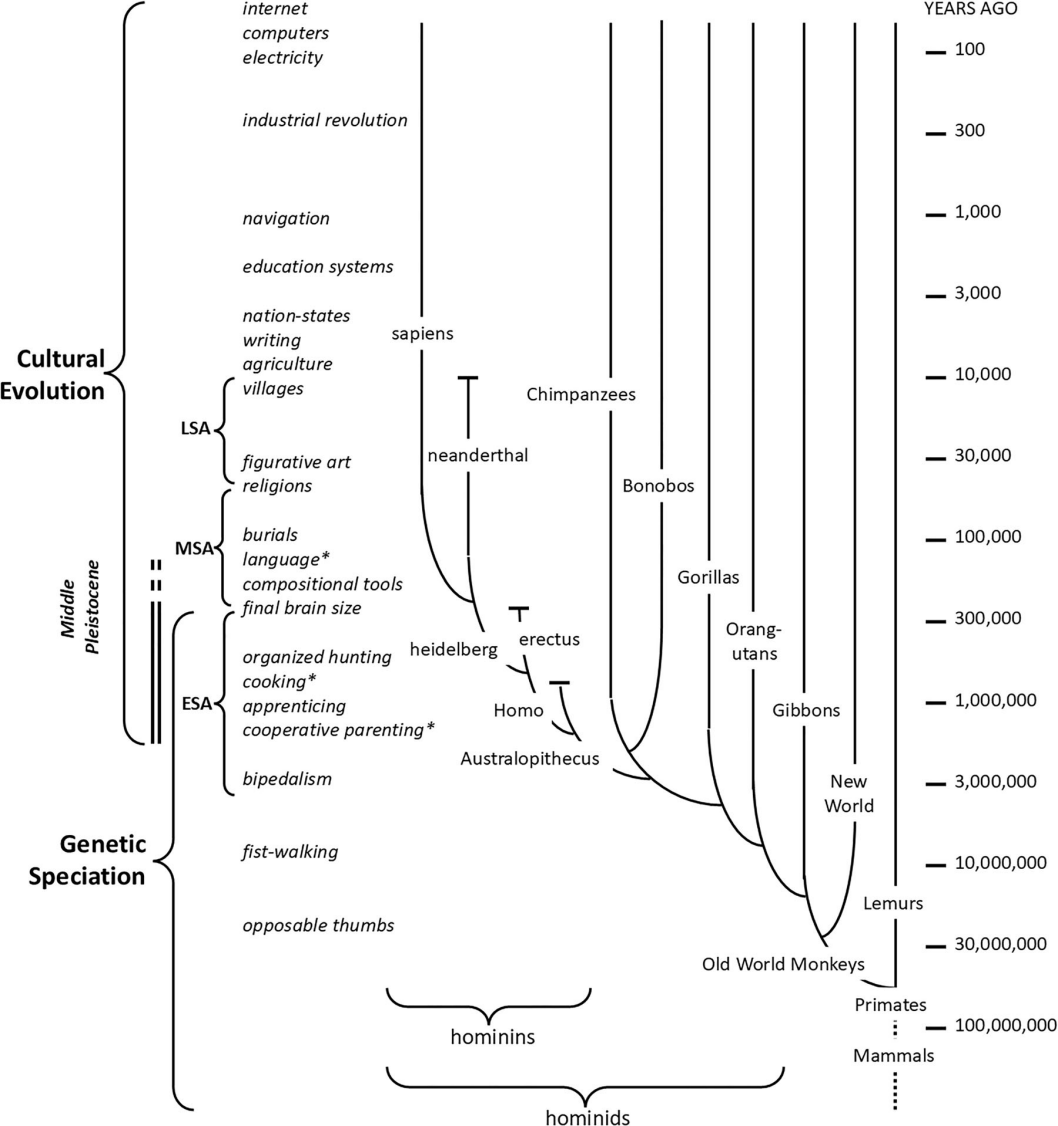
Major evolutionary steps in hominoid evolution on a log time axis with approximate appearance of associated hominin attributes [9]. The attributes associated with Homo sapiens (* discussed in depth below) are largely the product of cultural evolution during the Early, Middle and Later Stone Ages (ESA, MSA, LSA), which enabled large‐scale socioeconomic organization as the LSA progressed. Hypothetically, this accelerated cultural evolution was enabled by concurrent genetic and cultural changes (vertical double lines) that simply downregulated the instinctual behavior that dominates all other animals, rather than the sudden appearance of new genetic traits.

Human uniqueness manifests as the rapid and cumulative evolution of highly variable social behaviors and technological capabilities in the genus Homo and its several species [7, 8], a few of which are identified along the logarithmic timescale in Figure 1. Both the rapid appearance and the variable occurrence and distribution of most of these attributes are inconsistent with genetic evolution. They are the consequences of cultural evolution, in which individuals and societies invent rather than inherit such attributes [10, 11]. But something must have changed genetically that either permitted or perhaps forced humans to invent cultures. This paper hypothesizes that the enabling phylogenetic steps in human evolution included downregulation of instinctive behaviors that were limiting the potential of prior and concurrent physical and mental enhancements such as increased cerebral development, articulate oropharynx, upright posture and dexterous hands. Importantly, such downregulation would not require new genes and it would not necessarily give rise to consistent behaviors or cultures. Human societies forced to invent and maintain social organizations and technologies would be subject to the temporal and spatial vagaries of climate and geography [12]. Their cultural traits would appear, disappear and reappear, confounding the paleoanthropological record [4].

First we consider how genetically specified physiology affects and is affected by behavior and interpersonal relationships. Then we consider how downregulation of some of these effects both enables and forces behaviors to shift from instinctual to learned. Particular attention is paid to behaviors that facilitate that learning (cooperative parenting in Figure 1) and that support metabolically the mental power implicit in the cerebral enlargement that defines *Homo sapiens* phylogenetically (cooking in Figure 1). The burden of learning these behaviors and reconciling them with residual instincts leads to consideration of how the separate mechanisms of genetic downregulation of instincts and their cultural replacement may have reinforced each other during the Middle Pleistocene. In particular, the development of language can be seen as a necessary response to a loss of instinctual behavior as well as an enabler of the extraordinary diversity and dynamic competition that we see between human cultures and among individuals within a culture.

Genetic changes that down‐regulate pre‐existing genes and instinctive behaviors rather than new genes are less likely to be recognized as such, but they may provide a particularly powerful accelerator for gene‐culture coevolution (see Box 1 for definitions). Forward relationships from genetic evolution to behavior are the subject matter for the somewhat contentious field of “evolutionary psychology”, with sociopolitical implications that are often fought out in the lay press [13, 14]. The mechanisms and models of reciprocal coevolution are more complex and have only recently been recognized, particularly in human evolution but also in other social animals including nonhuman primates, cetaceans and birds [15, 16].

Box 1 **Key definitions.**

Behavior: actions intended to advance goals of an organism

Instinct: innate and stereotyped behavior in a given situation

Downregulation: decrease in the strength of an input‐output relationship

Culture: a set of learned behaviors, values and beliefs that are passed down within an organized social group

Cultural evolution: the process of cultures changing over time

Gene‐culture coevolution: a virtuous cycle in which changes associated with each type of evolution facilitate changes in the other (negative interactions also occur but are not considered here)

## 2 The Relationship of Genes to Behavior

It is useful to distinguish between the evolution of the body and the evolution of behavior [17]. The physical structure of the body is determined primarily by genes; behavior emerges from the interactions between the body and information about the environment in which the body happens to find itself [18]. All spiders make silk and most spin webs that have highly species‐specific designs [19], but they appear to learn the behavioral patterns of their prey [20]. Nest‐building birds instinctively build nests with the form associated with their species, but they may learn to refine their designs, for example to accommodate novel sites and building materials [21]. Humans are particularly in need of shelter but must learn or invent designs and constructions; these will be limited by their individual mental capacity and available resources.

Organisms that evolve more complex nervous systems can accomplish more sophisticated behaviors, increasing their survival and propagation in novel environments. Thus, behaviors might be driven (if not entirely determined) by genetically specified physical circuits similar to the spider or bird (herein called instinctual) and/or they might incorporate or depend on memory of the consequences of previous behaviors or examples (herein called learned). Many behaviors reflect a combination of instinctual (genetic) motivation and learned refinement [22]. Furthermore, the learning processes themselves depend on genetically specified interneuronal connections and intracellular mechanisms. Disentangling these relationships for any specific observed behavior tends be difficult [23], but across animal phylogeny, behaviors tend to shift from predominantly instinctual to predominantly learned.

Instinctual behavioral patterns have the advantage of providing an immediate, reliable repertoire of useful behaviors, but they do not provide a way to take advantage of new opportunities. As pointed out by J. Mark Baldwin in the early days of evolutionary theory, a single organism with a new, potentially useful mutation of its body must survive and reproduce for that mutation to persist and evolve further [24, 25]. That individual will be unlikely to survive if its behaviors are fixed genetically and optimized for a different body or environment. On the other hand, learning behaviors from trial‐and‐error experience takes time and exposes all individuals of that species to risks that reduce their fitness in the familiar environment.

Genetic evolution provides mechanisms for the development and maturation of the information processing parts of the body, including both the endocrine system and the nervous system. Their mechanisms determine the range of behaviors that might be learned while delimiting the behaviors actually expressed to avoid those that might be immediately fatal either to the individual or to the local breeding population in the case of social species. Tension between instinctual and learned behaviors is inevitable because each may interfere with the other. The evolutionary success of any mutation that affects either type of behavior will depend on how it interacts with the other.

The evolution of social species in which individuals play specialized roles is particularly fraught with potential conflicts between instinctual and learned behavior. If the specializations are genetically specified within fixed subgroups (as in workers, drones and queens in bees), evolution will be constrained within a given breeding population by the complex interactions among the specializations. If the specializations arise in individuals as a consequence of their behavioral experiences and innate physical heterogeneity, then the emergent behaviors are less constrained, presenting both opportunity and danger to the breeding group. The group might be better able to adapt to a novel environmental challenge or the appearance of a mutant individual but it might also self‐destruct in its usual environment.

The spectacularly rapid evolutionary success of *Homo sapiens* can be attributed to an extreme ability to take advantage of changed circumstances, including the changes resulting from their own actions. Not only do humans learn to refine their instinctual behaviors during their lives (as do some other animals), they also create cultures that invent, promulgate and accumulate new behaviors [26]. The beginnings of such dependence on learned rather than instinctual behaviors can be seen in the extant primates most closely related to *Homo sapiens* such as orang‐utans [27]. By comparing behavioral variability to genetic variability, Van Schaik found that variations in behaviors such as tool use for extracting seeds from fruit were largely due to the inventions of hominoid cultures. Similar genetic analyses of variations in vocal calls distinguish reliably between the learned calls of some bird species and the genetically determined calls of frogs.

Many of the highly labile social behaviors invented by *Homo sapiens* have precedents in other species [28], but an individual species usually expresses only one or a small subset of these behaviors, while humans are capable of them all. For humans the specifics of how and when to select a mate, how to and who should raise offspring, what to eat, how to govern, and many other social behaviors are mostly (but not entirely) highly variable inventions rather than instincts. Furthermore, two breeding groups of humans in similar circumstances may choose radically different social structures and then compete against each other for dominance. Such cultural inventions can be developed and then spread and dominate within a few generations, enabling a form of cultural competition and evolution that occurs at lightspeed compared to the genetic evolution of instinctual behavior [11]; note logarithmic timescale in Figure 1. The exponential growth in the size of cultures at the end of the LSA would accelerate the appearance [29] but not necessarily the acceptance [30] of cultural changes. Such cultural evolution requires not just the enlargement of brain capacity for invention but also the downregulation of instinctive behaviors that would otherwise constrain the acceptance of those inventions.

## 3 Examples of Downregulated Neural Circuits

Downregulation means a decrease in the strength of an input‐output relationship (a gain in engineering terms), such as the behavioral response to a stimulus. It can be accomplished via a variety of loci and mechanisms in a neuroendocrine system. The sensors for the stimulus might be reduced in their numbers or sensitivity; the signal from the sensors might lead to a weaker output; or the generator of the response might be weakened. Genes responsible for these various steps might be mutated to a less effective form or gene expression may be modulated epigenetically. Neurons and endocrine cells receive chemical inputs (transmitters, hormones, metabolites) and convert them into internal electrical signals that, in turn, cause the release of chemical outputs (transmitters or hormones). The strength of the response to one input can be downregulated by another input that shifts the electrical signal away from the value at which the cell generates an output. The nervous system is organized into circuits that typically include chains of interneurons, each of which receives many inputs that shift its electrical state in one or the other direction.

The layered structure of information processing in the nervous system provides a substrate in which older, more preprogrammed subsystems tend to persist but are modulated by more recently evolved subsystems for learned behavior. The phylogenetically older endocrine and autonomic nervous systems that we associate with instinctive behaviors are reciprocally connected to the more recent central nervous systems of vertebrates. Hormones released by the former modulate the activity of the latter [31], providing a basis for instinctually driven behavior. But the central nervous system also modulates the endocrine and autonomic nervous systems, both by generating neurotransmitters that have their own hormonal effects and by direct innervation of endocrine, digestive and immunological subsystems by the hypothalamus and vagus nerve, among other pathways [32]. Clinical dysfunctions related to mental stress include, for example, hypothalamic amenorrhea [33] and changes in both adrenocorticosteroids and growth hormones [34]. It is difficult to attribute causality during normal function in such feedback systems, which are usually considered from the perspective of maintaining homeostasis or circadian rhythms. The following examples from the sensorimotor system provide clearer examples of the general potential for behavior to evolve phylogenetically by dynamically downregulating (i.e. modulating) the contributions of older, more automatic pathways.

In mammals the spinal cord preserves reflex connectivity from somatosensory afferents to motoneurons similar to that found even in insects, but it is routed through interneurons that can be biased on or off by descending control from the brains of vertebrates [35]. Shifts in the electrical biasing of such interneurons can cause a behavior that is automatic in one species to require learning and voluntary control in another [36]. The spinal cord contains central pattern‐generation circuits that specify the sequences of muscle activation required for locomotion [37], enabling a newborn colt to walk almost immediately. Humans start to crawl automatically but then suppress crawling to learn bipedal locomotion slowly and as a novel behavior that is better suited to their anatomy [38]. Learning – i.e. “inventing” – how to walk enables humans (but not horses) eventually to learn to play soccer, which requires coordination patterns that are not available in genetically specified central pattern generators.

The hardwired, vertebrate subsystem in the phylogenetically older midbrain enables automatic, fast and accurate acquisition of sensory targets in extrapersonal space (e.g. a frog catching a fly with its tongue), but it can be gated on or off or given imagined targets by the phylogenetically recent forebrain (cerebral cortex) in mammals [39, 40]. Failure to suppress fast, reflexive midbrain function in superior colliculus may account for attention deficit hyperactivity disorder (ADHD) in humans [41]. ADHD is but one example of the general phenomena of impulsivity, which are known to vary among but be generally weaker in humans than other species under comparable circumstances [42].

More complex behaviors such as foraging and mating determine reproductive success but pose immediate risks to the individual. The more highly regulated they are by instincts, the more likely they are to succeed under usual circumstances but to miss opportunities offered by changing circumstances. One source of change would be increased climate variability, which appears to be correlated with major genetic developments in hominin evolution [12]. Instinctual behaviors are driven by emotions and desires mediated by the phylogenetically old limbic system, also known as the “reptilian brain”. Culturally learned behaviors are driven by the cerebral cortex, which accounts for almost all increasing brain size (relative to body size) in the sequence of species within the genus Homo [43, 44]. The connections between the two are reciprocal, allowing instinctual drives to influence behavior but also allowing cortex to down‐regulate the limbic system.

Successful instincts of social mammals tend to include at least some environmental contingency rules, such as how to change breeding patterns if the relative proportions of males to females changes [18]. This route to complexity is limited, however. The increasingly layered structure of the evolving mammalian brain preserves and modulates robust instinctual behaviors in the lower layers but makes it increasingly difficult for genetic evolution to construct novel instincts there. In addition to the inherent complexity of higher behaviors, they must interact with the other previously acquired instincts and the subsystems that generate them. Humans have the intelligence to invent such contingent strategies, but their implementation depends also on genetic downregulation of instincts that might compete with them.

## 4 Examples of Downregulated but Residual Instincts

One way to identify instinctual mechanisms that have been downregulated is to examine the behaviors for which humans are more flexible than other animals. Interestingly, most research on such behaviors has focused on identifying vestiges of the genetically programmed mechanisms that still influence those behaviors [45] rather than considering the positive consequences of their downregulation relative to other animals. It is as if science is still looking for evidence in support of Darwin's *Descent of Man* rather than moving on to the consequences.

Human ability to evolve cultures and build large societies has been attributed to the genetic evolution of new instincts such as “pro‐social motives” [46, 47]. In extant humans, however, the correlations between genetic polymorphisms and behavior are quite weak, while the effects of cultural upbringing are much stronger [48]. Here we consider instead why hard‐won evolutionary instincts that originally enabled increasingly complex social behaviors of vertebrates had to be downregulated genetically to enable the much more rapid cultural evolution of *Homo sapiens*. The resulting cultural behaviors could themselves facilitate learned downregulation of the instinctive pathways via the descending pathways from the forebrain – a coevolution. This process would iterate cyclically, accelerating both genetic and behavioral heterogeneity.

The lives of virtually all multicellular animals are governed by a very large number of instincts that can be triggered by various input signals and mediated by a wide range of neural and endocrine mechanisms as noted above. I have chosen here to focus on pheromonal inputs and hormonal mechanisms because these are known to be strong in other animal species and present but relatively weak (i.e. downregulated) in humans.

### 4.1 Interpersonal Behavior Driven by Pheromones

Pheromones are chemicals secreted by one animal that are transmitted to other animals as smells that affect their behavior [49]. Research on human pheromones has been controversial and often inconclusive, at least in part because humans have sparse connections to and weak perception by underdeveloped olfactory systems [50].

The pheromones that influence mate selection and bind mothers to their infants [51] are just some of many olfactory cues that persist but have become faint in humans [52]. The hormones that drive virility in males and fertility in females have strong odors, which is why the enzyme that converts androgens to estrogens is called aromatase [53]. They are easily detectable in sweat and urine and drive reproductive behavior in most species. They seem to change human perceptions of attractiveness [54] and actual sexual behavior in both men [55] and women [56], but only modestly (in both senses of the word).

Fear almost instantly changes the nature and odor of human sweat. Dogs are good at detecting this in humans [57]. So are humans, even when they don't know it. Sweat collected from other humans under fearful conditions (skydiving) elicited changes in the metabolic activity of parts of the brain associated with fear, even when the subjects reported no perceptible difference in smell [58]. Overt behavioral effects of fear pheromones have been well‐documented in various mammals, but little is known about their exact chemical composition in humans [59].

Distinctive body odor results from a combination of kinship genes, hormonal state, dietary habits, hygiene and the microbiome of the skin [60]. One of the cues that promotes friendship relationships among humans appears to be that they smell similarly [61]. Body odor was likely one of many cues that promoted the instinctive tribalism that was useful for competing bands of hunter‐gatherers [62], but it is generally counterproductive for multi‐ethnic nation‐states that are a recent and uniquely human invention. Conversely but more subtly, parental heterogeneity in their immune systems is beneficial for their offspring and is associated with heterogeneity in pheromones that women find attractive in men [63]. Vaginal secretions depress the motility of sperm from males with similar immune genes, and such couples have fewer children [64].

Pheromones drive the extraordinarily diverse behaviors of various species of ants [65]. They determine the various roles of individuals in the colony, they distinguish members of the colony from intruders and they paint a target on the nests of competing ant colonies and species. They mark the trails that allow one scout to summon thousands of its brethren to partake of a windfall. Subtle changes in these pheromones and their behavioral effects account for the great variety of social organization and daily behavior seen in the > 13,000 different species of ants. Most ant species form individual colonies that aggressively protect their territories and war frequently with the adjacent colonies, something like the small, inbred human tribes that have survived in remote places like the Amazon river basin and the mountain jungles of New Guinea [66]. In his book *The Social Conquest of Earth*, the naturalist Edward Wilson detailed the many parallels between the social behavior of ants and humans [67]. Argentine ants, an invasive species that is dominant in the US Southwest, appear to have lost the tendency to develop unique pheromones when they establish new colonies. This results in “supercolonies” that extend over hundreds of miles and millions of nests that live in peace with each other [68], something like the human citizens of a large, multi‐ethnic nation. This more gregarious culture was enabled for both species – ants and humans – by a downregulated component of their instinctual behaviors.

Anyone who has ever walked a dog knows that they depend on scent to identify other dogs and their territories [69]. Dogs are a large step up from ants in that they can associate smells with their rich, multi‐sensory experiences of individual dogs (and humans) that they have encountered. But they are a step below humans in being unable willfully to ignore olfactory cues.

Genetic evolution left humans with a notably impoverished sense of smell. We start with unusually few olfactory receptors (less than 5% of a dog's or even a rabbit's) connected to a relatively small patch of brain [70]. Decreases in the size of the primate olfactory processing centers occurred as the visual system came to dominate both the growing intracranial volume and diurnal behavioral patterns of primates, so it is difficult to disentangle cause and effect [71, 72]. Genes supporting pheromone sensing, however, “have been impaired and removed from functional constraints since shortly before the separation of hominoids and Old World monkeys ~23 million years ago, and … the random inactivation of pheromone receptor genes is an on‐going process even in present‐day humans” [73]. Within the hominins, Neanderthal and Denisovan DNA show losses of olfactory receptor genes that are different from each other and from *Homo sapiens* [71], suggesting a more complex pattern of evolutionary exploration of fitness. While loss of pheromone receptors would enable and perhaps require compensatory cultural behaviors to emerge, that process takes time. The individual carrying such a mutation must survive for that mutation to persist and provide a basis for further gene‐culture coevolution.

Olfaction depends also on the availability and concentration of the odorants. Upright posture takes human noses away from the ground and the genitals, where the most interesting and useful odors tend to be found. This is perhaps why evolution endowed humans with hairy armpits that become smelly as the rising sex hormones in adolescence interact with the mostly odorless secretions of young children [74]. Nevertheless, loss of most body hair and a fondness for immersing ourselves in water makes us less smelly than the rest of the apes. Modern culture reinforces this by regular bathing, scented soaps, deodorants and perfumes. Thus both genetic and cultural evolution have conspired to reduce the salience of olfactory cues, probably because they would otherwise drive the instinctive behaviors that humans must suppress as they develop large, multi‐ethnic societies.

Systematic study of the odorant receptor genes across extant and extinct populations of hominins is increasingly feasible [75]. If coupled with better understanding of the various instinctive behaviors triggered by the pheromones, this could provide tests of the hypothesis that the unique cultural evolution of *Homo sapiens* was enabled by the genetic downregulation of those instincts.

### 4.2 Child Rearing Driven by Hormones

Vertebrates exhibit a great range of parenting behaviors, including species in which neither or only the female or only the male or both provide physical protection or nourishment or both [76]. These species‐specific behaviors are largely instinctual and driven primarily by hormones released during mating, pregnancy and lactation. Human relationships are complicated by the fact that successful human reproduction requires an unprecedented duration of parenting compared to other animals. Selecting a mate whose appearance and courtship behavior suggests that he or she could be an effective parent is not the same as actually getting him or her to do so after a demanding and smelly infant arrives. Another layer of motivating instincts is required.

Instinctive behaviors of many infant animals trigger useful instinctive behaviors in their parents. A human infant reacts to a hovering human face with a reflexive smiling response that adults find irresistible [77], mediated by a serotonin‐oxytocin circuit through the adult hypothalamus [78]. Both the sound of crying and the tactile sensations of suckling induce oxytocin and prolactin release, reinforcing lactation in mothers [79]. Fathers also respond to infant crying with increased testosterone and prolactin production [80]. Recently, lower expression of the oxytocin receptor gene in some human superpopulations has been associated with relative looseness of their social norms, an example of downregulation in gene‐culture coevolution [81].

Pregnancy and delivery are accompanied by massive shifts in hormones that prepare the female body for motherhood but also have deep psychological effects on attitudes and behaviors [82]. Their partners certainly perceive the physical and behavioral changes and they probably detect the associated pheromones subliminally. Behavioral changes in mothers binding with their infants appear to be correlated with persistent decreases in the volume of certain areas of cerebral cortex, which may be one of the many results of the hormonal and other physiological changes associated with pregnancy and lactation [83]. Perhaps more surprisingly, similar brain shrinkage occurs in fathers [84]. New fathers generally experience a decrease in their testosterone levels that is associated with greater caregiving. In turn, male infants raised with strong paternal caregiving tend to larger such effects when they become fathers [85]. This suggests an intergenerational transmission of parenting style that facilitates the development of cultures that are appropriate for the environment in which humans find themselves. In a high‐risk environment, males are more likely to propagate their genes successfully by promiscuously inseminating as many females as possible, a behavior that is known to be promoted by high testosterone levels. In a more stable environment, a man would do better to form a lasting relationship with a fertile woman and a nurturing relationship with prospective heirs.

Long before there were physicians or midwives or nannies or self‐help books, hominins were successfully reproducing based on instincts alone, just like all other animals. The unique thing about humans is that we can choose to ignore our instincts, which suggests the need for the professional advice. We can be convinced by external advice that infants must be picked up immediately if they cry or left to cry themselves out, regardless of our instincts. We can override protective instincts and abandon our offspring or engage in physical abuse of our spouse and children. We can force ourselves to leave our infants in the hands of strangers because daycare facilitates providing our offspring with material goods and the educational opportunities required for success in a modern society.

Cultural norms for prolonged familial nurturing represent an obstacle to the instinctive dissolution of some parent‐child bonds that is required for the formation of new and independent family units. The surges of hormones associated with puberty drive aggressive behaviors that are distressing to parents but attractive to potential mates [86]. Such disruptive instincts serve an obvious purpose but advanced societies mandate a greatly extended adolescence to acquire the advanced skills required to succeed. Humans can decide to keep their postadolescent children under the same roof long after residual instincts encourage their eviction to form new families. Even unrelated members of the community tolerate disruptive behavior and cooperate to facilitate the vast amount of social learning required for children to become competent in the complex culture that the community has accumulated [87].

Until recently, parental decisions were considered no one else's business. It is only in the past few decades that society has felt not only empowered but even obligated to intervene [88]. This is paradoxical in the context of contemporary societal decisions to step back from long‐standing interference in life‐style choices such as homosexuality, adultery, contraception and abortion. How might we explain this? Since at least Babylon, children were perceived to be assets of their parents (Code of Hammurabi, #209, 210). Most obviously, they were the precious vessel for their parents’ genes. In the absence of social insurance schemes, they were the investment whereby parents could hope for a longer life and more pleasant death. As fertility rates plummeted and investment in public education surged in industrialized societies, however, children suddenly became an asset of society at large. There are other primates that care for their offspring communally [89], but they do so instinctively, not politically.

The decisions that remain delegated to parents depend to some extent on subjective personal instincts rather than objective cultural practices. The hormones associated with biological motherhood exert powerful and (usually) useful effects on the mother's judgment and interactions with her offspring. Biological fathers and adoptive parents experience some similar effects but they are inevitably weaker and conflated with mating rather than parenting [90]. Societies are now experimenting with progressive policies such as equal parental leave from employment for mothers and fathers, but their uptake has been slow and limited [91]. It remains to be seen how much of these behavioral tendencies are cultural effects that society can change, at least gradually, and how much they reflect residual animal instincts driven by our virtually immutable genes. But the mere fact that such experiments are possible is evidence of the profound downregulation of instinctual behavior in favor of cultural invention.

## 5 Examples of Compulsively Learned Behaviors

Animals that must learn from experience with their environments require motivation to expend effort and take risks while doing so. Young cats learning to hunt will spend hours “playing” with captured prey, presumably to learn their moves and refine their own responses [92]. Cats do so when satiated, suggesting that what we call “curiosity” is a preprogrammed tendency of higher nervous systems to seek out and respond to random reinforcement schedules [93]. Learning something as complex as social behaviors requires even more complex and lengthy trial‐and‐error practice, during which the immature human is relatively ineffectual and vulnerable. A curiosity drive seems to motivate all humans when young and many throughout life. It is particularly likely to manifest itself in those activities that are important for survival and procreation but for which the genetically defined instincts available to other animals have been downregulated or even suppressed. An anthropologist from another world comparing humans to other primates would immediately notice that humans have an almost obsessive interest in activities that other primates mostly ignore or take for granted.

### 5.1 Mating Behavior

All social animals have a large stake and take great interest in who mates with whom, but the mating behavior itself is stereotyped. The development of novel courtship and copulation strategies is an important mechanism of speciation, leading to great variety in the animal kingdom but little or none within a given species. Jared Diamond explored the evolution of human sexuality, identifying animal precedents for many human behaviors (and their culturally defined perversions) while noting some that are rare or novel among other animals, including our closest phylogenetic relatives [94]. Perhaps more remarkable, but still unremarked upon [95], is the human obsession with studying the courtship and copulation strategies of other humans. Pictorial and sculptural representations of humans engaged in sexual activity are among the earliest artefacts of just about every civilization unearthed by archeologists. More modern forms of communication such as written language, the printing press, still photography, motion pictures, videotape and the digital internet can all trace their widespread promulgation and commercial success to sexual stories and images [96]. If such material is intended for sexual arousal, it falls under the definition of pornography.

The *Kamasutra* (circa 225 CE) contains much advice on how to live but became infamous in sexually repressed Western cultures for its depictions of sexual positions. Gutenberg's invention of the printing press in Europe (anticipated by centuries in China) premiered with *Biblia Sacra* in 1455, which includes the sexually explicit “Songs of Solomon”. This was followed by popular printed editions of Chaucer's *The Canterbury Tales* (1476 from manuscript in 1400) and Boccaccio's *Il Decameron* (1492 from manuscript in 1353). About one‐third of the tales in each include explicit erotica, usually illicit but generally romantic [97, 98, 99].

Photographic technology became practical in 1839 and was almost immediately employed in the production and sale of pornographic images [100]. Edison's Kinetoscope and successors such as the Mutoscope used film strip technology to provide peep shows, often pornographic. These evolved into the longer “blue movie” (the actual title of a 1969 film by Andy Warhol). Obsessive interest in the mating habits of other humans was captured in the title of the 1967 Swedish erotic hit film *I Am Curious (Yellow)*. Sony's video cassette recorder in 1971 facilitated the expansion of the clandestine “stag film” industry [101]. The internet made all this once‐illicit pornography and exhibitionism much more accessible. A substantial percentage of internet traffic remains pornography of one sort or another [102].

Downregulation of instincts might lead to such compulsive interest in sexuality because modern humans do not instinctively know what constitutes acceptable sexual relations in their own or other cultures. If humans just needed to learn how to perform a sexual act, however, this plethora of sexual material would be overkill. Sexual practices might differ among cultures, but it wouldn't take much effort to learn a specific behavior that was the norm for a given culture. In fact, many of the highly diverse practices outlined by Jared Diamond coexist within a culture, if not overtly then certainly clandestinely. The downregulation of instincts that linked sexual behavior to fertility and procreation allowed such behavior to be repurposed for social organization, so its importance goes beyond procreation. The high level of heterogeneity in the physical, hormonal and behavioral attributes of individuals within a society gives rise to a similar level of heterogeneity in the collection of mating rituals and sexual practices that coexist within each society. As each individual approaches puberty, he or she must learn about and select from this sexual smorgasbord, a daunting task. One strategy would be to settle for the first thing that worked reasonably well. That shortcut has been largely foreclosed by the extended adolescence mandated by advanced economies, where it is impractical or even illegal to form a stable union between two underage sexual partners.

### 5.2 Food Preparation

All social animals have a large stake and take great interest in how food is distributed and shared among individuals in breeding groups, but the selection of food is driven largely by subconscious nutritional needs and adventitious availability. Lots of animals spend hours perfecting their behaviors for foraging and hunting, but once a food item has been obtained, it is consumed with little preparation or ceremony and usually immediately. Long after their nutritional needs are satisfied, however, humans devote a huge amount of effort to learning about (and frequently inventing) novel food combinations and preparation methods, i.e. cooking. Humans need to learn food preparation because they do not instinctively know what to do with the ingredients available in their own or other cultures. We speak of humans as omnivores (an instinct of some animal species) but in fact humans are “oligovores”. Each human culture develops complex recipes involving a relatively small percentage of available foodstocks and often disdains (e.g. insects, dogs, horses) and/or prohibits (e.g. Islamic halal, Jewish kosher, Jainism) other ingredients.

As hominid brains and their metabolic demands grew in response to the opportunities offered by enhanced cognition and communication [103], the extraction of energy from the cumbersome and inefficient digestion of raw foods became a limiting factor in human evolution. What we call foods are the bodies of other animals (meat) and the aspirational progeny of plants (seeds and fruits), all of which evolved to resist the disaster of being digested. They require a great deal of energy to break down into usable sources of energy, making them inefficient and less attractive as foods [104]. Cooking with fire lets humans supply that energy from external sources, mostly the burning bodies of yet other plants and animals (wood and oil). Finding and learning to combine and use more nutritious raw foods is also a culturally transmitted aspect of food preparation. Cooking facilitated the final increases in brain size and various changes to the digestive tract that accompanied the appearance of *Homo sapiens* at the beginning of the MSA, an example of gene‐culture coevolution [105]. Cooking became the continuing and competitive elaboration of fundamental inventions of human civilization such as control of fire. But cooking is a learned rather than instinctive behavior. Mastering it requires trial‐and‐error experimentation that is often wasteful in the moment but rewarding in the long term, making it a target for our curiosity drive.

Communications media have been heavily invested in cooking as well as pornography. There are recipes recorded on clay tablets from ancient Mesopotamia circa 1700 BCE [106]. Printed cookbooks were prized possessions of affluent Europeans in the Renaissance [107, 108] and hand‐written ones are still passed from mothers to daughters everywhere. Newspapers and magazines devote many column‐inches to reviewing the latest restaurants and food fads. Cable television has whole channels devoted to cooking shows and competitions. Cooking and recipe apps are popular on cellphones and personal computers. Food “selfies” are pervasive; Google just returned 633,000,000 search results.

Cooking and food preservation required large and phylogenetically recent behavioral changes that were driven by human invention (fire, agriculture, animal husbandry) rather than genetic evolution, but the results are so powerful that they have driven at least some simple genetic selection even within the relatively short existence of *Homo sapiens*. Perhaps the best known of these relates to lactase, the digestive enzyme for the predominant sugar in milk. The extended childhood of humans, which is necessary to learn non‐instinctive social behaviors, necessitated a mechanism to get young children to stop nursing, which imposes a metabolic demand on mothers that suppresses their fertility. The earliest humans stopped producing lactase and could no longer digest milk after early childhood. The inventions of animal husbandry and preservation of milk as yoghurt, butter and cheese drove a genetic reversal of lactase suppression in Euroasian subpopulations [109]. Other dietary inventions were facilitated by selection and drift in the microbiome of the gut, which performs much of the digestive work and has its own signaling mechanisms with the human nervous system [110].

Cooking and food preservation may also drive the cultural half of a gene‐culture coevolution with olfaction. Genetic analysis of olfactory receptors for food odorants indicates that *Homo sapiens* has developed a wider range of genetic variants than early hominins, most of which result in downregulated sensitivity [75]. Organized agricultural commerce and novel recipes would make olfactory instincts for food selection less important, leading to genetic variation, while reduced sensitivity would increase tolerance to novel food smells in evolving cultures.

Food selections and preparation methods are distinguishing features of human cultures. Infants acquire preferences for the food dishes that they have experienced [111] and their microbiomes adapt to deal efficiently with them [112]. Customary diets may be enshrined in religious laws that help to distinguish competing cultures and their tribal members as well as promoting healthful or efficient behaviors. How well such dietary customs interact with the availability and economic cost of their ingredients in the society's environment becomes another aspect of a culture's fitness.

## 6 Mutual Reinforcement Between Genetic and Cultural Evolution

Genetic evolution is usually studied by comparing the morphology and behavior of variously related species separated by hundreds or thousands of generations. Intermediate forms must have existed but they tend to disappear as useful, stepwise adaptations accumulate. The rate of such adaptations and the appearance of new species both accelerate rapidly when changes in the environment present large opportunities with little competition, i.e. punctuated equilibrium [113]. The hominin explosion of the Middle Pleistocene that is still being elucidated by paleontologists and geneticists may reflect evolution punctuated by changes in the environmental circumstances wrought by the hominins themselves. It would be further accelerated by genetic exchange among incompletely speciated hominins [114]. If cultural evolution was enabled by genetic evolution that downregulated some instinctual behaviors, then this would also create an opportunity for acceleration of genetic evolution. The examples above of rapid and functionally related genetic increases of endocranial volume [115] and back‐and‐forth changes of digestion are consistent with such opportunities.

The relationship between the genetic and cultural evolution of *Homo sapiens* can be viewed from the perspective of Aristotle's and Plutarch's causality dilemma – which came first: the chicken or the egg? We now understand that a random genetic mutation results in a change of phenotype, so the egg comes first [116]. But we also understand that the survival of the chicken determines whether that mutation persists and, hence, whether other mutations can be added to it to extend the incipient speciation. A genetic mutation that downregulates an evolved, hence presumably useful, instinctive behavior necessarily reduces the fitness of that individual but might be accommodated if it enables a latent ability to learn a more effective behavior. The learned behaviors of individuals carrying that mutation might then come to dominate the culture, reducing the fitness cost of further mutations that further downregulated instinctual behaviors [67]. Such iteration would have been particularly strong during the period of overlap between the last major steps in genetic evolution and the first steps in cultural evolution (2 to 0.3 million years ago in Figure 1, corresponding to Early Stone Age).

Perhaps the most important cultural engine for accelerating iterative cycles of gene‐culture coevolution is extended childhood. Young humans lacking instinctive behaviors to survive are sheltered by their parents and the community at large while they learn how to survive and procreate in the culture into which they are born [87, 117]. For modern *Homo sapiens*, extended parenting behavior may be a combination of instinctive protection of offspring carrying our genes plus culturally learned behaviors that greatly outlast and may even conflict with residual genetic instincts for hominin parenting. Such conflicts might then limit the development or successful promulgation of cultures.

### 6.1 Coevolution of Complex Spoken Language

The invention of complex language may be another example of accelerated coevolution. In the absence of instinctively understood social messages based on pheromones and hormones, individuals who could better convey their intentions verbally would have an important advantage. So, too, would individuals who could better understand complex instructions for the new necessity of food preparation. Cultural complexity would be limited by the ability of mature individuals to convey cultural norms to juveniles, a challenge that was addressed by the human invention of complex language [118]. Put simply, *Homo sapiens* had more to talk about than other primates.

The phylogenetic evolution of the human vocal tract has been inferred from subtle clues in the basal cranium, which, unlike the larynx, tends to be preserved in the fossil record [119]. Fundamental changes to the aerodigestive tract (relationships among mouth, trachea and esophagus) that enabled humanlike speech appear to be unique to *Homo sapiens*. The specific speech sounds that humans can make, however, depend on details of the oropharynx (lips, tongue, hard and soft palate, alveolar ridge supporting teeth) that are so recently evolved that they remain heterogeneous among individuals and unevenly distributed geographically. Such anatomical details may provide a basis for the heterogeneous and geographically distinctive phonemes associated with different languages [120]. The parts of the central nervous system that subserve spoken language and the genes associated with its evolution are also just starting to be elucidated [121, 122].

Punctuated genetic evolution over ~100 ka at the beginning of the MSA (dashed double lines in Figure 1) could account for rapid enhancement of both the physical and mental capacity for spoken language. Complex language, in turn, facilitated the elaboration of cultures that could replace instincts, thereby facilitating the further downregulation of genetically programmed instincts that constrained the further development of cultures – a virtuous cycle. Studies that relate genes to structure to function in speech are in their infancy, but they could eventually provide insights into the gene‐culture coevolution of uniquely human attributes.

Vocalization patterns in animals have provided a basis for studies of gene‐culture coevolution, but generalizations across widely separated species are fraught. Cultural transmission of learned birdsong has been associated with accelerated speciation because it rapidly divides the species into smaller breeding groups subject to gene drift [15]. Language differences tend to isolate and separate human cultures, but this appears to have had only a marginal effect on gene flow [123]. Most (but not all) birds learn their songs once during a critical period of development, but humans can acquire new languages at any age. Mating in birds is essentially consensual whereas much historical human mating was not, particularly when intercultural.

### 6.2 Polygenicity Leads to Behavioral Heterogeneity

The most complex social animals benefit from heterogeneity in the physiognomy and behavioral tendencies of individuals within a breeding population [124, 125], who can take up specialized roles most suited to their individual attributes. Such heterogeneity arises from the random combinatorial effects of the many genes that contribute to those attributes.

Studies of heritability usually assume that the genes responsible for a given phenotype have additive effects. Various types of epistatic interaction between genes are known to confound this assumption [126]. Such epistatic effects seem particularly likely for behavioral traits that depend on endocrine and nervous systems whose structure and function emerge from experience during development rather than only from genes that directly specify that structure and function [127]. A very large number of genes encode the rules for those developmental effects, making the effects of any one of them highly dependent on the other genes and on the unique experiences of the individual possessing the complete genotype. This reduces heritability of behavioral phenotype from these genes, which also favors increasing variance in such genes [128]. The downregulation of relatively fixed, genetically‐specified behavioral traits in favor of learned behaviors further weakens the heritability of behavioral phenotype, further increasing genetic variability and unpredictable epistatic interactions.

Because behavioral phenotype is highly polygenic and epistatic and those genes are highly heterogeneous, behavior does not “breed true” [129]. This leads to unusually high levels of heterogeneity in human traits such as personality type and various forms of intelligence. Such heterogeneity persists even in small bands of interbreeding individuals that might otherwise be subject to rapid selection and gene drift [130]. Not coincidentally, it provides and sustains the diverse social substrate required for the invention of many, very different patterns of social behavior, i.e. cultures. Any culture will empower some and disenfranchise other personality types, guaranteeing a subclass motivated to invent a new and distinct culture.

### 6.3 Implications for Further Human Evolution

I propose that the unique attributes of *Homo sapiens* reflect downregulated instinctual behavior. Initial genetic downregulation enabled cultural invention of more adaptive learned behavior, which in turn reduced the importance and genetic stability of instincts. This coevolution would be expected to iterate and accelerate. If true, then the relative contributions of genetic versus cultural evolution must shift inexorably from the former to the latter [131]. Downregulation of a process has an obvious boundary when the process no longer contributes to the attributes of the species. Cultural evolution involves the addition of new layers of behaviors, with no obvious upper bound. Furthermore, as the log scale in Figure 1 indicates, cultural evolution involves mechanisms that are vastly faster than genetic evolution, which complicates studies and models of interactions between the two. It is convenient to assume that cultural evolution, like genetic evolution, is inexorable and amoral. While that fits most of history, it ignores more recent human efforts to protect both cultural and species diversity for their own sakes.

There is no going back to instinct. The well‐being of modern human societies depends completely on the cultural inventions of large‐scale government, finance, trade and technology that are anathema to the unsuppressed instincts of earlier hominins. Furthermore, *Homo sapiens*’ instincts have become too weak to sustain any level of social organization, even that of those earlier hominins. Humans have transcended the treadmill of genetic evolution and replaced it with the treadmill of cultural evolution. The former comes with a zoo of examples demonstrating how it works; the latter comes without instructions.

## Data Availability

Data sharing not applicable to this article as no datasets were generated or analysed during the current study.
